# Altered Expression Levels of MMP1, MMP9, MMP12, TIMP1, and IL-1*β* as a Risk Factor for the Elevated IOP and Optic Nerve Head Damage in the Primary Open-Angle Glaucoma Patients

**DOI:** 10.1155/2015/812503

**Published:** 2015-05-11

**Authors:** Lukasz Markiewicz, Dariusz Pytel, Bartosz Mucha, Katarzyna Szymanek, Jerzy Szaflik, Jacek P. Szaflik, Ireneusz Majsterek

**Affiliations:** ^1^Department of Clinical Chemistry and Biochemistry, Medical University of Lodz, 90-647 Lodz, Poland; ^2^Department of Cancer Biology, AFCRI, Perelman School of Medicine, University of Pennsylvania, PA 19104, USA; ^3^Department of Biochemistry and Molecular Biology, Hollings Cancer Center, Medical University of South Carolina, Charleston, SC 29425, USA; ^4^Department of Ophthalmology, Medical University of Warsaw, 03-709 Warsaw, Poland

## Abstract

The aim of presented work was to analyze the impact of particular polymorphic changes in the promoter regions of the -1607 1G/2G* MMP1*, -1562 C/T* MMP9*, -82 A/G* MMP12*, -511 C/T* IL-1β*, and 372 T/C* TIMP1* genes on their expression level in POAG patients. Blood and aqueous humor samples acquired from 50 patients with POAG and 50 control subjects were used for QPCR and protein levels analysis by ELISA.* In vivo* promoter activity assays were carried on HTM cells using dual luciferase assay. All studied subjects underwent ophthalmic examination, including BCVA, intraocular pressure, slit-lamp examination, gonioscopy, HRT, and OCT scans. Patients with POAG are characterized by an increased mRNA expression of* MMP1*,* MMP9*,* MMP12*, and* IL-1β* genes as compared to the control group (*P* < 0.001). Aqueous humor acquired from patients with POAG displayed increased protein expression of MMP1, MMP9, MMP12, and IL-1*β* compared to the control group (*P* < 0.001). Allele -1607 1G of* MMP1* gene possesses only 42,91% of the -1607 2G allele transcriptional activity and allele -1562 C of* MMP9* gene possesses only 21,86% of the -1562 T allele. Increased expression levels of metalloproteinases can be considered as a risk factor for the development of POAG.

## 1. Introduction

Primary open-angle glaucoma (POAG) is one of the leading causes of irreversible blindness. There were 60.5 million people with open-angle glaucoma (OAG) and angle-closure glaucoma (ACG) in 2010, increasing to 79.6 million by 2020. Women comprised 55% of OAG, 70% of ACG, and 59% of all glaucoma in 2010. Bilateral blindness were present in 4.5 million people with OAG and 3.9 million people with ACG in 2010, rising to 5.9 and 5.3 million people in 2020, respectively [1, 2]. There are two main theories of the development of POAG: biomechanical and ischemic. The elevated intraocular pressure (IOP) is considered to be the main risk factor in biomechanical theory of POAG development [3]. Elevated IOP results from dysfunctional aqueous drainage through the trabecular meshwork (TM) [4]. An elevated plaque-like material and altered remodeling process within the TM have been linked to the IOP increase in POAG patients [5]. In recent years, many studies have linked shifting the equilibrium between extracellular matrix (ECM) synthesis and breakdown within the TM with the apoptosis of retinal ganglion cells (RGC) [6–8]. Furthermore, alterations of the ECM within the juxtacanalicular (JXT) portion of the TM have been found to be a primary pathophysiologic association with POAG. Thus, proteins known to regulate ECM equilibrium might strongly influence IOP levels. The role of matrix metalloproteinases (MMPs) in pathogenesis of POAG has been strongly suggested [9–11]. Alterations of the MMPs-mediated and endogenous ECM turnover regulate the outflow resistance. Therefore, ongoing ECM turnover is necessary for homeostatic maintenance of the IOP.

Thus, the aim of this study was to evaluate expression level of MMP1, MMP9, MMP12, and TIMP1 in blood and aqueous humor of POAG patients compared to control group of people without any type of glaucoma diagnosed. Moreover, we compared expression levels with polymorphic variants previously determined by our team [12]. Beside the main MMPs and their inhibitor TIMP1 analysis, IL-1*β* levels were examined as this interleukin is reported to cause overexpression of some MMPs [13].

## 2. Material and Methods

### 2.1. Subjects

We enrolled 50 POAG patients for the presented study. All POAG patients (*n* = 50) and controls (*n* = 50) were matched on age and sex (no differences were calculated, *P* > 0.05) (Table 1). Controls were the people not diagnosed with any type of glaucoma and their visual acuity ranged from 20/20 to 20/30. All studied subjects underwent ophthalmic thorough examination; HRT and OCT scans were conducted. After the diagnosis of POAG in accordance with the guidelines of European Glaucoma Society (*Terminology and Guidelines for Glaucoma IInd Edition, Dogma, Savona 2003, Italy*), blood was collected. The patients with POAG at the time of sample collection were prior any topically antiglaucoma treatment. Aqueous humor (HA) samples were taken during cataract surgery (controls) or trabeculectomy (POAG). Aqueous humor (100 *μ*L) was withdrawn through an ab externo limbal paracentesis site with a 27-gauge needle on a tuberculin syringe. Meticulous care was taken to avoid touching intraocular tissues and to prevent the contamination of aqueous samples with blood. The samples were immediately (after mixing with protein inhibitors cocktail) frozen in liquid nitrogen and stored at 80°C. Medical history was obtained from all subjects, and no one reported present or former cancer or any genetic disease. Patients were excluded from the study if they were subject to any of the following conditions, which could possibly interfere with the results of the study: use of eye drops, any ocular surgeries or laser treatments performed in the past in the eye from which the specimens were to be collected, present or prior treatment with glucocorticosteroids or immunosuppressive therapy (if these treatments had not been stopped at least 1 year before the surgery and collection of specimens), use of nonsteroidal anti-inflammatory drugs (with the exception of low-dose aspirin, which had to be stopped 7 days before the surgery and collection of specimens), and prior and concurrent systemic antibiotic treatment during the last 7 days before the start of the study. The study was reviewed and approved by the local Ethics Committee (Permission number RNN/468/10/kB) and met the tenets of the Declaration of Helsinki. Written consent was obtained from each patient before enrolment in the study.

### 2.2. Cell Cultures

Primary human trabecular meshwork cells (P10879) were purchased from Innoprot (Derio, Bizkaia, Spain). Cells were isolated from juxtacanalicular and corneoscleral region of human eye. Culture flasks were coated with poly-L-lysine (2 *μ*g/cm^2^). Cells were cultured in Fibroblast Basal Medium (P60108, Derio, Bizkaia, Spain) supplemented with 2% fetal bovine serum (FBS), Fibroblast Growth Supplement (FGS), and antibiotics (100 U/mL penicillin G, 100 mg/mL streptomycin, and 250 ng/mL fungizone) at 37°C in a humidified 5% CO_2_ atmosphere. When the cultures reached confluence (about 90%), cells were suspended by 0.025% (w/v) trypsin/0,5 mM EDTA and seeded onto 6-well plate (4.0 × 10^6^ cells/mL; 4 mL for each well) the day before the transfection for the measurement of luciferase activity.

### 2.3. RNA Isolation and QPCR

Blood samples from 50 POAG patients and 50 healthy control subjects were collected in 3 mL EDTA tubes and mixed with RNA later buffer (Thermo Scientific, Waltham, MA, USA). RNA was isolated from peripheral blood lymphocytes using isolation kit: QIAamp RNA Kit (Qiagen, Chatsworth, CA, USA) according to the instructions provided by the manufacturer. The analysis of the expression level by QPCR was performed using ready-made kits Brilliant SYBR Green QPCR II kits (Agilent Technologies, Santa Clara, CA, USA) according to the protocol supplied by the manufacturer. The reaction was carried out using the Stratagene Mx3005P QPCR system (Agilent Technologies, Santa Clara, CA, USA) supplied with the corresponding analytical software. Primers were designed with ProbeFinder online tool (Roche, Indianapolis, IN, USA) so that they are in adjacent exons in order to exclude the formation of the product from the genomic DNA (Table 2).

**Figure 1:**
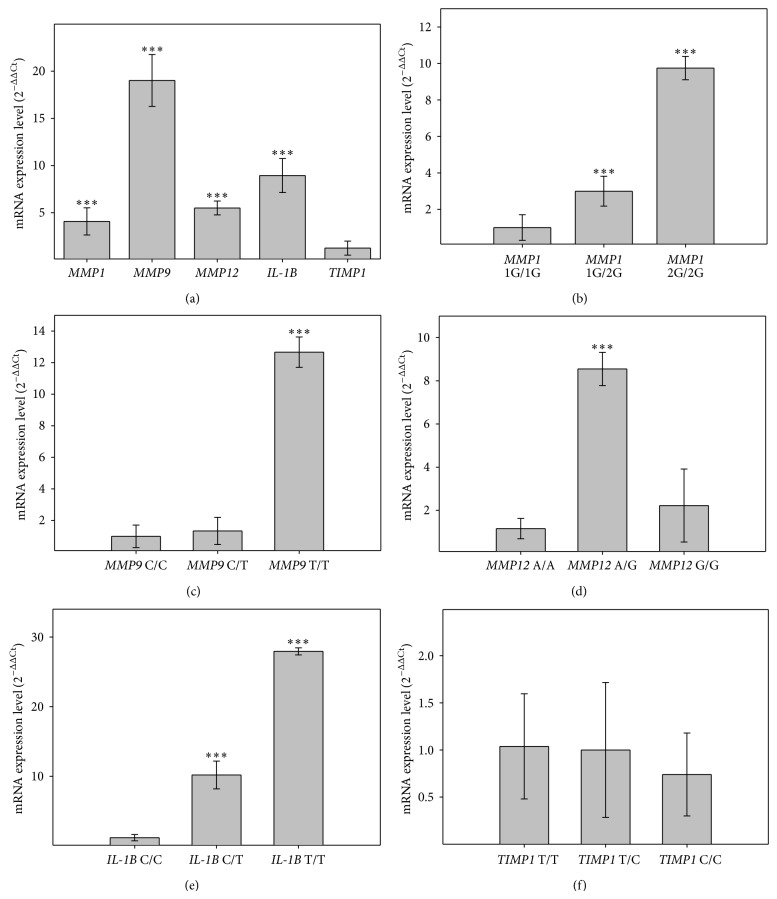
Analysis of the mRNA expression levels. (a) Overall expression level of in the POAG patients compared to control group without any type of glaucoma (value after subtraction of the control group presented as fold change 2^ΔΔCt^); expression levels with regard to genotype in POAG patients group: (b)* MMP1*; (c)* MMP9*; (d)* MMP12*; (e)* IL-1β*; (f)* TIMP1*. Data presented as fold change compared to the wild type genotype (whose expression was taken as 1). Error bars represent standard deviations (SD). ^∗∗∗^ *P* < 0.001. (*n* = 50). There was no difference in genotypes distribution between patients with OAG and control subjects (*P* > 0.05). MMP1: 1G/1G = 17; 1G/2G = 17; 2G/2G = 16 MMP9: C/C = 22; C/T = 23; T/T = 5 MMP12: A/A = 23; A/G = 23; G/G = 4 IL-1*β*: C/C = 17; C/T = 17; T/T = 16 TIMP1: T/T = 17; T/C = 17; C/C = 16.

Immediately after the isolation of RNA was digested with DNase (Promega, Madison, WI, USA) in order to exclude any contaminating DNA, all RNA samples were diluted to equal concentration, and then RNA was transcribed into cDNA using AffinityScript QPCR cDNA Synthesis Kit (Agilent Technologies, Santa Clara, CA, USA) according to the manufacturer's instructions. For QPCR reactions, 1 ul of obtained cDNA was used, and each sample was prepared in triplicate. The annealing temperature for all primers was 60°C. In the analysis of the expression level of the parameter 2^ΔΔCt^ calculated from the formula wherein the parameter Ct is the threshold cycle value obtained for a given sample, ΔCt was the difference in Ct values of interested gene or particular genotype and the Ct of reference gene* GAPDH *and ΔΔCt was the difference between the expression level (ΔCt) in POAG patients compared to controls (Figure 1(a)) or between particular genotype within the POAG patients group (Figure 1).

### 2.4. ELISA Tests

HA samples were thawed on ice and 100 *μ*L was used for the analysis. Control subjects were selected from the group of individuals without POAG. MMP1, MMP9, MMP12, and TIMP1 proteins levels were measured by enzyme immunosorbent assays (ELISA) (RayBiotech, Inc., Norcross, GA, USA) and IL-1*β* levels (Diaclone SAS, Besancon Cedex, France), which were performed according to the manufacturer's instructions. Specifically, assays were solid-phase immunoassays derived from the direct sandwich technique, which uses biotinylated anti-MMP monoclonal antibody (MAb), streptavidin coated microstrips, and HRP labeled anti-MMP MAb. Protein levels were determined by standard spectrometry on Synergy HT microplate reader (BioTek, Winooski, VT, USA).

### 2.5. Dual Luciferase Assay

The* MMP1* promoter region (containing -1607 1G allele or 1607 2G allele) stretching from -1682 to +152 and* MMP9* promoter region (containing -1562 C allele or -1562 T allele) stretching from -1664 to +128 were cloned into the pGL4.11-basic vector containing the luciferase reporter gene (Promega, Madison, WI, USA). The following constructs were produced: pGL-1G-MMP1, pGL-2G-MMP1, pGL-C-MMP9, and pGL-T-MMP9 (*MMP1* 2G and* MMP9* T alleles were generated using the QuikChange II site-directed mutagenesis kit (Stratagene)). Cloning was performed using the following set of primers:

 MMP1 left: TCTTCTGAGCTCCAAGTGTTCTTTGGTCTCTG;
 MMP1 right: TCTTTACTCGAGTGTGCATACTGGCCTTTGTC;
 MMP9 left: TAAGCAGGTACCTGGGCAGATCACTTGAGTAGA;
 MMP9 right: TAAGCACTCGAGTCTTCCCTGGAGACCTGAGA

containing, respectively,* Sac*I,* Xho*I,* Kpn*I, and* Xho*I restriction sites which were used in PCR product and subsequent vector digestion. Constructed plasmids after transformation of* E. coli* DH5*α* were isolated using QIAGEN plasmid DNA preparation kits and cotransfected (along with pRL-TK, an internal control with renilla luciferase gene) into HTM cells (Innoprot, Derio, Bizkaia, Spain) using a TurboFect Transfection Reagent (Thermo Scientific, Waltham, MA, USA). For each reaction, 2 *μ*g of each plasmid was used. Cells were placed on 6-well plates. To assess promoter transcriptional activity, cells after 48 h were subjected to dual luciferase activity assay system (DLR, Promega, Madison, WI, USA) according to the manufacturer's protocol. The firefly luciferase levels (FLA) were standardized for renilla luciferase levels (RLA) as an internal control for each sample. Assay was quantified using Synergy HT microplate reader (BioTek, Winooski, VT, USA). Data was shown as RLU calculated as FLA/RLA obtained from 3 independent experiments (separate transfections, cells from 5th passage).

### 2.6. Statistical Analysis

Comparisons between mean values for each group were carried on using* t*-test. The statistical calculations used STATISTICA 6.0 software (StatSoft, Tulsa, OK, USA). The statistical significance of the mean values between the two sets of data Student's* t*-test was used. Results are presented as mean ± standard deviation (SD).

## 3. Results

### 3.1. mRNA Levels Analysis

mRNA expression level analysis (with respect to previously published genotyping data [12]) showed a nearly 5-fold increase in the level of* MMP1* gene expression in POAG patients group compared to control subjects (*P* < 0.001) (Figure 1). Moreover, nearly 3-fold expression increase has been observed for the homozygous genotype -1607 2G/2G compared to -1607 1G/2G heterozygous genotype and 9-fold compared to 1G/1G homozygous genotype within a group of POAG patients (*P* < 0.001) (Figure 1).* MMP9* gene expression analysis showed nearly 20-fold increase in POAG patients compared to the control group (*P* < 0.001) (Figure 1) and 9-fold and 12-fold increase in the expression level for -1562 T/T genotype as compared to the -1562 C/T and -1562 C/C genotypes, respectively, within a group of POAG patients (*P* < 0.001) (Figure 1).* MMP12* gene displayed over 5-fold expression increase in POAG patients compared to the control group (*P* < 0.001) (Figure 1). It has shown nearly 9-fold increase in the expression level of -82 genotype A/G genotype compared to -82 A/A in POAG patients group (*P* < 0.001) (Figure 1). Interestingly, we did not observe an increase in the expression level for -82 G/G genotype.* IL-1β* gene was 10-fold overexpressed within the group of patients with POAG when compared to the control group (*P* < 0.001) (Figure 1). Results showed more than 25-fold increase in the expression level of -511 T/T genotype when compared to -511 C/C within a group of POAG patients (*P* < 0.001) (Figure 1). There was no statistically significant change in the level of* TIMP1* gene expression in the study group when compared to the control group (Figure 1).

### 3.2. Protein Expression Level

**Figure 2:**
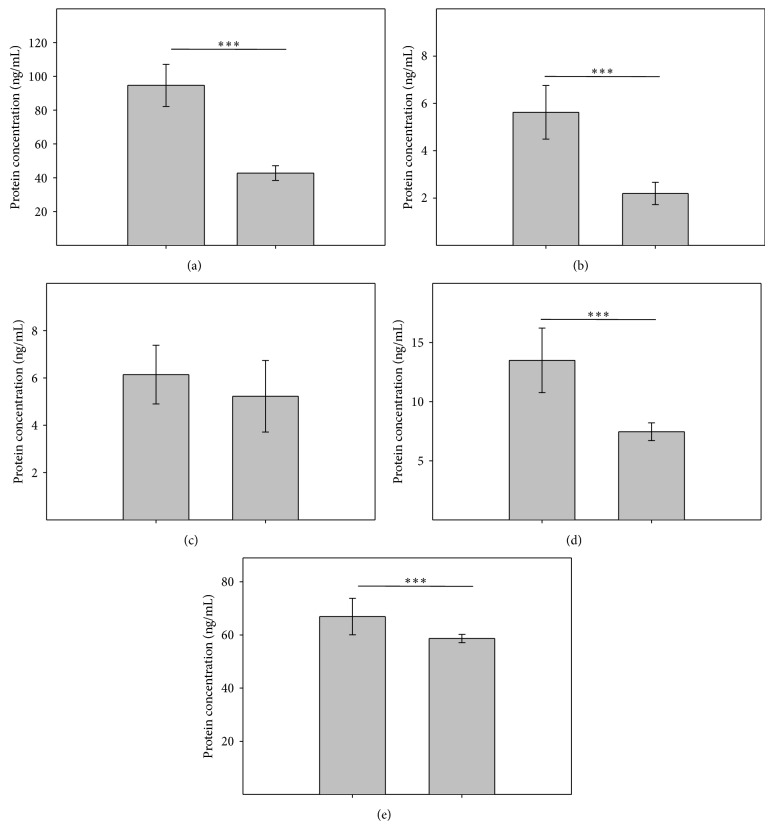
Analysis of protein expression levels for MMP1 (a); MMP9 (b); TIMP1 (c); MMP12 (d); and IL-1*β* (e) in POAG patients (1) aqueous humor compared to the control group without any type of glaucoma (2). Error bars represent standard deviations (SD). ^∗∗∗^ *P* < 0.001. (*n* = 50).

The MMP1 analysis showed 2.2-fold (*P* < 0.001) increase in protein concentration in the aqueous humor obtained from POAG patients when compared to the control group (94.64 ng/mL versus 42.75 ng/mL) (Figure 2). MMP9 had 2.5-fold increase (*P* < 0.001) in the aqueous humor of POAG patients when compared to control subjects (5.62 ng/mL versus 2.10 ng/mL) (Figure 2). We observed almost 2-fold increase (*P* < 0.001) in the concentration of MMP12 in the aqueous humor from patients with POAG compared to the control group (13.49 ng/mL versus 7.46 ng/mL). Comparing the levels of IL-1*β*, an increase in protein concentration in the aqueous humor of POAG patients compared to control subjects (66.93 pg/mL versus 58.68 pg/mL, *P* < 0.001) was observed. Similar to mRNA experiments, analysis of TIMP1 protein showed no statistically significant increase in the protein concentration in the aqueous humor from patients with POAG compared to control subjects (6.14 ng/mL versus 5.23 ng/mL) (Figure 2).

### 3.3. MMPs Promoter Activity Analysis

**Figure 3:**
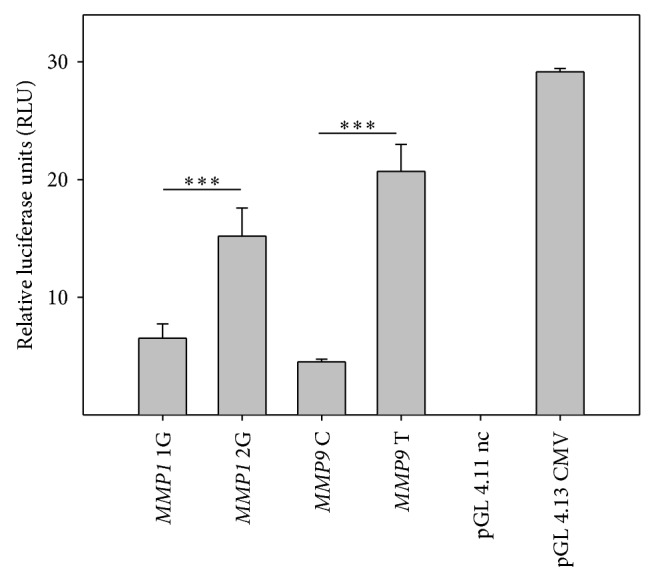
MMPs promoter activity. Plasmid constructs harboring:* MMP1* 2G: insertion of additional guanine in position -1607 (2G) in* MMP1* gene (pGL 4.11* MMP1* 2G);* MMP9* T: change in position -1562 from C to T in* MMP9* gene. pGL 4.11 nc: negative control (promoterless); pGL 4.13: positive control (CMV promoter). Data presented as relative luciferase units RLU calculated as FLA/RLA of the sample. Error bars represent standard deviations (SD). ^∗∗∗^ *P* < 0.001. Data from three independent experiments with independent transfections (*n* = 3).

We utilized dual luciferase assay to assess the influence of the -1607 1G to -1607 2G allele change on the level of MMP1 and -1562 C to -1562 T allele on MMP9 expression. Results showed an increase in relative luciferase units (RLU) for the construct harboring allele -1607 2G of* MMP1* gene compared to -1607 1G (15.21 RLU and 6.52 RLU resp.) and for the allele -1562 T of* MMP9 *gene compared to -1562 C (20.69 RLU and 4.52 RLU, resp.) (Figure 3). The allele -1607 1G of* MMP1* gene possess only 42,91% of the -1607 2G allele transcriptional activity and allele -1562 C of* MMP9* gene possess only 21,86% of the -1562 T allele transcriptional activity.

## 4. Discussion

POAG is a major cause of irreversible vision loss [1, 2]. The outflow of aqueous humor from the anterior chamber of the eye can be disturbed by increased amount of residual material from the remodeling of collagen meshwork. This is usually associated with elevated IOP in POAG patients [5]. Many researchers suggest the role of matrix metalloproteinases in the pathogenesis of POAG [9–11]. Hernandez et al. identified astrocytes as key cell types involved in the process of progressive damage to the optic nerve. These studies showed that astrocytes can be activated by an increased IOP [14, 15]. Yan et al. demonstrated that these activated astrocytes are responsible for the production of matrix-degrading enzymes that affect the process of remodeling [16]. However, the path from the reconstruction of the TM to the death of RGC is not yet completely understood. MMPs play an important role in the remodeling of extracellular matrix components, which affects the behavior of the integrity of the tissue. Therefore, in the present study we analyzed the mRNA and protein expression of the following polymorphic variants* MMP1* -1607 1G/2G, -1562 C/T* MMP9*, -82 A/G* MMP12*, -511 C/T* IL-1β,* and 372 T/C* TIMP1* in MMPs and* IL-1β* genes. Previously, we demonstrated strong relation between aforementioned polymorphisms and increased risk of POAG development [12]. Many recent studies have shown that significantly altered levels of MMP in aqueous humor were obtained from glaucomatous patients [9–11]. Rönkkö et al. showed significant excess of the MMP1 over TIMP1 in samples taken from patients with POAG [17]. An increased expression of MMP1 in human optic nerve head astrocytes POAG has also been observed in study performed by Agapova et al. [18]. Mossböck et al., however, did not find any correlation of MMP1 with POAG patients from Austria [19]. Our analysis of mRNA confirmed the above hypothesis, showing an increase in the expression level of -1607 2G/2G genotype compared to the 1G/1G genotype within the group of patients with POAG (*P* < 0.001). Furthermore, we compared retinal nerve fiber layer (RNFL) thickness for each allele observed (previously published results) [12]. In the presence of 2G MMP1 overexpressing allele RNFL was significantly decreased compared to normal expression (1G allele) (0.178 ± 0.08 versus 0.321 ± 0.10, resp., *P* < 0.001). We observed similar relationship for the MMP1 protein levels, which also displayed an increase in concentration in the aqueous humor of patients with POAG compared to the control group. Previously, only direct effect of the increased MMP9 expression level on the loss of retinal ganglion cells was reported [20]. The starting point for this study was the observation that MMP9 is constitutively expressed at low levels in RGC cell layer [20]. Guo et al. found that the growth of MMP9 activity in RGC apoptotic cells is accompanied by reduced amount of laminin, thus indicating the increased degradation of ECM [21]. Our data confirmed those findings showing more than 12-fold increase in expression level -1562 genotype T/T genotype as compared to the -1562 C/C within a group of POAG patients. Previously published genotyping results along with presented analysis of mRNA suggests that the presence of T allele has an adverse effect both on the risk of POAG as well as clinical parameters. We observed decreased RNFL thickness related to -1562 T allele compared to -1562 C (0.192 ± 0.08 versus 0.169 ± 0.05, resp., *P* < 0.001). The increase in the amount of mRNA was consistent with subsequent analysis of protein levels of MMP9, which showed a 3-fold increase in patients with POAG aqueous humor compared to control subjects of POAG patients. Results showed over a 5-fold increase in the expression of the* MMP12* gene in patients with POAG compared to the control group. An 8-fold increase in mRNA expression level of -82 genotype A/G genotype compared to -82 A/A in patients with POAG patients was shown. Analysis of protein levels showed a similar correlation. There was almost 2-fold increase in the concentration of MMP12 protein in the aqueous humor collected from patients in comparison with POAG aqueous humor collected from the control group. In accordance with the results of the central nervous system, it is suggested that a rapid induction of proinflammatory cytokine IL-1*β* may play an important role in neuronal degeneration. Zhang and Chintala suggest that the induction of IL-1*β* in the optic nerve damage promotes retinal MMP9 by increasing the synthesis of [22]. Chua et al. reported increased levels of IL-9, IL-10, and IL-12 in patients diagnosed with glaucomatous changes [23]. Studies by Mookherjee et al. indicate that the region comprising IL-1 gene affects the pathogenesis of POAG [24]. Chua et al. showed significant differences in the profile of cytokines present in the aqueous humor of patients with POAG compared with age-matched controls [23]. These studies have shown that the increase of MMP9 concentration can be the reaction for overexpressed cytokine IL-1*β*. We observed similar correlation for IL-1*β* and POAG occurrence. The mRNA expression level of -511 T/T* IL-1β* genotype was more than 3.5-fold increase compared to -511 C/C genotype within a group of patients with POAG. Increased level of IL-1*β* in POAG corresponded to the observed MMP1 overexpression. Surprisingly, it was shown that the correct value of the rim surface in the optic disc and RNFL thickness is associated with the presence of -511 T/T* IL-1β* genotype in patients with POAG. These data suggest that IL-1*β* gene may be considered as a risk factor for POAG, which, however, is not associated with subsequent progression rate of the disease. Other molecular mechanisms need to be studied in order to fully elucidate the IL-1*β* involvement in the pathogenesis of POAG.

To investigate the promoter-dependent altered expression mechanism, dual luciferase assay was utilized for promoter activity analysis. Results showed that -1607 1G to 2G MMP1 allele change and -1562 C to T MMP9 allele contribute to increased protein expression. The data indicate that the presence of additional guanine within the -1607 2G allele can cause formation of PEA3 consensus sequence binding site of AP-1 in the promoter region [25, 26]. The presence of that site can result in a significant increase in the affinity of the promoter sequence for transcription factor AP-1, significantly raising the level of expression. Therefore, it can be assumed that the signal transduction (mainly through IL-1*β* signaling) influences the expression of MMP1 2G variant results in a significant expression increase and elevation of POAG development risk [27]. The influence of the -1562 C/T polymorphism on* MMP9* gene transcription therefore remains unstudied. There are suggestions indicating that C to T change increases the expression by lowering the binding affinity, causing corepressor to release from the DNA strand. Our results seems to be in agreement with those shown by Tseng et al. that IL-1b promotes corneal epithelial cell migration by increasing MMP-9 expression mainly through AP-1-dependent pathways [13].

In conclusion, altered levels of MMP1, MMP9, MMP12, and IL-1*β* can have significant effect on the development of POAG. Increased expression is connected with particular changes in promoter sequences of the genes. It can be considered as a risk factor for the development of primary open-angle glaucoma. Moreover, our previously published genotyping data together with the findings of the presented work indicate that increased expression of MMPs has also an influence on the clinical parameters of optic nerve. Thus, we postulate that the negative impact of MMP overexpression can be associated with mechanism of MMP-dependent increased IOP, ultimately leading to RGC apoptosis and probably direct MMP-dependent apoptosis of RGC in the OHN. Our results might help to finally elucidate the role of MMPs in the pathogenesis of POAG. The findings suggest that complex changes in the MMP-TIMP balance and altered MMP activity in aqueous humor may promote not only abnormal matrix remodeling characteristic of POAG but also RGC apoptosis which may be causally involved in the pathogenesis of POAG.

## Figures and Tables

**Table 1:** The clinical characteristic of open-angle glaucoma (POAG) patients and control group.

	Parameter	Patient groups	Control group
Percentage of the group	Gender male/female	38%/62%	52%/48%
	Hypertension^a^	54%	70%
	Low blood pressure^b^	40%	29%
	Vascular disease	33%	49%
	Diabetes mellitus type 2^d^	17%	55%

Mean ± SD	Age (years)	73 ± 10	64 ± 1 6
	Intraocular pressure, IOP (mmHg)	13.2 ± 2.9^∗^	11.9 ± 1.9
	Cup disk ratio (c/d) right eye/left eye	0.72 ± 0.16/0.70 ± 0.16	PNM^c^
	Rim area (RA) right eye/left eye	1.19 ± 0.43/1.25 ± 0.37	PNM^c^
	Retinal nerve fiber layer (RNFL) right eye/left eye	0.18 ± 0.08/0.23 ± 0.22	PNM^c^

^a^Systolic pressure > 140; diastolic pressure > 90 mmHg.

^b^Systolic pressure < 90; diastolic pressure < 60 mmHg.

^c^PNM: Parameter not measured.

^d^No differences observed for the studied proteins expression level when DM and non-DM subjects were compared in each group (*P* > 0.05).

^∗^After the treatment: all of the patients enrolled to this study had increased IOP above 21 with the average of 23.3 ± 1.9 measurement taken during first appointment prior to antiglaucoma treatment.

**Table 2:** The primer sequences used for mRNA expression level analysis by real-time QPCR.

Gene	Primer sequences	Product size
*MMP1 *	F: 5′-CAGAGATGAAGTCCGGTTTTTC-3′	76 pz
	R: 5′-GGGGTATCCGTGTAGCACAT-3′	

*MMP9 *	F: 5′-GAACCAATCTCACCGACAGG-3′	67 pz
	R: 5′-GCCACCCGAGTGTAACCATA-3′	

*MMP12 *	F: 5′-AGTTTTGATGCTGTCACTACCG-3′	64 pz
	R: 5′-CACTGGTCTTTGGTCTCTCAGAA-3′	

*IL-1β*	F: 5′-TACCTGTCCTGCGTGTTGAA-3′	76 pz
	R: 5′-TCTTTGGGTAATTTTTGGGATCT-3′	

*TIMP1 *	F: 5′-GAAGAGCCTGAACCACAGGT-3′	77 pz
	R: 5′-CGGGGAGGAGATGTAGCAC-3′	

*GAPDH *	F: 5′-AGCCACATCGCTCAGACAC-3′	66 pz
	R: 5′-GCCCAATACGACCAAATCC-3′	
